# Honey bee colony performance affected by crop diversity and farmland structure: a modeling framework

**DOI:** 10.1002/eap.2216

**Published:** 2020-09-30

**Authors:** Juliane Horn, Matthias A. Becher, Karin Johst, Peter J. Kennedy, Juliet L. Osborne, Viktoriia Radchuk, Volker Grimm

**Affiliations:** ^1^ Helmholtz Centre for Environmental Research – UFZ Permoserstrasse 15 Leipzig 04318 Germany; ^2^ Environment and Sustainability Institute University of Exeter Penryn Campus Penryn Cornwall TR10 9FE UK; ^3^ Leibniz Institute for Zoo and Wildlife Research (IZW) in the Forschungsverbund Berlin e.V. Alfred‐Kowalke‐Straße 17 Berlin 10315 Germany; ^4^ Plant Ecology and Nature Conservation University of Potsdam Am Mühlenberg 3 Potsdam 14476 Germany

**Keywords:** *Apis mellifera*, BEEHAVE, colony viability, crop diversity, cropping system, decline, forage availability, forage gaps, honey bees, landscape generator, modeling

## Abstract

Forage availability has been suggested as one driver of the observed decline in honey bees. However, little is known about the effects of its spatiotemporal variation on colony success. We present a modeling framework for assessing honey bee colony viability in cropping systems. Based on two real farmland structures, we developed a landscape generator to design cropping systems varying in crop species identity, diversity, and relative abundance. The landscape scenarios generated were evaluated using the existing honey bee colony model BEEHAVE, which links foraging to in‐hive dynamics. We thereby explored how different cropping systems determine spatiotemporal forage availability and, in turn, honey bee colony viability (e.g., time to extinction, TTE) and resilience (indicated by, e.g., brood mortality). To assess overall colony viability, we developed metrics, *P*_H_ and *P*_P,_ which quantified how much nectar and pollen provided by a cropping system per year was converted into a colony's adult worker population. Both crop species identity and diversity determined the temporal continuity in nectar and pollen supply and thus colony viability. Overall farmland structure and relative crop abundance were less important, but details mattered. For monocultures and for four‐crop species systems composed of cereals, oilseed rape, maize, and sunflower, *P*_H_ and *P*_P_ were below the viability threshold. Such cropping systems showed frequent, badly timed, and prolonged forage gaps leading to detrimental cascading effects on life stages and in‐hive work force, which critically reduced colony resilience. Four‐crop systems composed of rye‐grass–dandelion pasture, trefoil–grass pasture, sunflower, and phacelia ensured continuous nectar and pollen supply resulting in TTE > 5 yr, and *P*_H_ (269.5 kg) and *P*_P_ (108 kg) being above viability thresholds for 5 yr. Overall, trefoil–grass pasture, oilseed rape, buckwheat, and phacelia improved the temporal continuity in forage supply and colony's viability. Our results are hypothetical as they are obtained from simplified landscape settings, but they nevertheless match empirical observations, in particular the viability threshold. Our framework can be used to assess the effects of cropping systems on honey bee viability and to develop land‐use strategies that help maintain pollination services by avoiding prolonged and badly timed forage gaps.

## Introduction

Honey bees (*Apis mellifera* L.) are a key pollinator of insect‐pollinated crops and wild plants, with overall insect pollination services being estimated to exceed US$ 153 billion in agricultural systems (Gallai et al. 2009). Thus, the ongoing substantial loss of managed honey bee colonies in Europe and the United States (Lee et al. 2015) is of particular concern. Keeping up with the rising demand for insect‐pollinated food production seems to be at high risk (Aizen et al. 2008).

As one important stressor, substantial losses of suitable habitats resulting from agricultural intensification and lack of floral resources as alternatives to crops have been assumed to crucially affect honey bee health and hive losses (Kleijn et al. 2006, Naug 2009, Vanbergen and the Insect Pollinators Initiative 2013, Clermont et al. 2015). Consequently, in many regions after a short period with ample amounts of nectar and pollen provided by mass‐flowering crops, there is a forage dearth in early spring and summer (Decourtye et al. 2010, Couvillon et al. 2014).

Apiculturists have long realized that landscape context is a critical factor for colony success (Sponsler and Johnson 2015). Temporary shortages in sufficient flowering resources, which are a typical phenomenon in spring and summer in many European agricultural landscapes, can strongly affect colony success (Decourtye et al. 2010). If such food shortages occur in a sensitive phase of colony development, i.e., when the colony is close to achieving maximum brood rearing and adult population size, the colony's resilience and survival capability are strongly impaired via cascading effects on life stages and tasks (Horn et al. 2016).

Systematically exploring in field experiments how landscape configuration and composition affect colony resilience and viability is not feasible (Henry et al. 2017). We therefore performed corresponding simulation experiments by using a two‐step modeling framework. First, we developed a landscape generator (NePoFarm) that generates and calculates, on a daily basis, the nectar and pollen supply of cropping systems scenarios that varied in landscape structure, and the crop species identity, diversity, and relative abundance. Second, we fed the resulting spatiotemporal data on nectar and pollen supply into the simulation model BEEHAVE (Becher et al. 2014) to assess honey bee colony performance under the different cropping system scenarios.

To limit the complexity of the scenarios to be explored, we ignored other stressors than those related to forage supply, such as pesticides, mite infestation, diseases, bad weather, or bad beekeeping practices. We also assumed that the probability of all flowering fields in the modeled landscape to be detected is 1.0, while in reality this might not be so. The model BEESCOUT (Becher et al. 2016) allows in principle to determine detection probabilities by explicitly simulating bee movement, but this would require data for parameterization that do not yet seem to exist. Consequently, with our settings the spatial arrangement of fields is probably less important than it might be occasionally in reality. Moreover, we only consider a single colony while in reality competition with other colonies and pollinators certainly plays a role. Our model predictions of colony persistence are thus relative, not absolute. They inform about the relative importance of forage availability in an otherwise perfect world. Interactions with other stressors remain to be explored in the future. The major aims of this study are to present our modeling framework for assessing honey bee colony performance for various cropping systems in a systematic way and to report our first generic findings. The framework will help to improve our understanding of how cropping systems could provide sufficient and sufficiently continuous nectar and pollen supply that meet the colony's requirements for ensuring viability and how temporary gaps in forage supply affect colony resilience. We applied our modeling framework to the most important European cultivated crops, asking the following questions: (1) What is the minimum diversity of cultivated crops required to meet a colony's food requirements for continuous food supply and thereby enable viability? (2) Does this minimum depend on the identity of the cultivated crops involved and their relative abundance? (3) How does farmland structure, i.e., spatial configuration and the size distribution of agricultural fields, affect colony viability?

## Methods

Due to the complexity of the question addressed and the tools used, considerable effort went into collecting data for parameterization and for analyzing and testing the model. Most details of the corresponding information are presented in Appendix S1 (Table 1).

**Table 1:** Overview of the content of the Appendix S1.

Location	Content	Description	Display items
Appendix : Section S1	model parameters, equations and assumptions	initial parameter settings, weather conditions suitable for foraging, most important model equations	2 tables 1 figure
Appendix : Section S2	farmland maps	how the two farmland maps we prepared to implement different cropping system scenarios s2‐1: r script “nepofarm” s2‐2: input file for nepofarm	1 table
Appendix : Section S3	crop species and their nectar and pollen data	source for selecting and parameterizing crop species (flowering periods, nectar and pollen parameters) as well as for the composition and abundances of the cropping systems considered	4 tables 12 figures
Appendix : Section S4	landscape generator NePoFarm to create scenarios as input files for the simulation model BEEHAVE	description of NePoFarm with links to corresponding R script and parameter file	4 tables 1 figure
Appendix : Section S5	*P*_H_/ *P*_P_: indices for comparing forage‐induced stress at the colony level	parameters and equations for calculating these indices	1 table
Appendix : Section S6	ranking farmland factors by mimicking a local sensitivity analysis procedure	description of sensitivity analyses and statistical procedures to evaluate their results	–
Appendix : Section S7	simulations—landscape settings	sensitivity analyses of nectar and pollen parameters, the distance and size of flowering fields, field size of monocultures, and of seminatural habitats	10 tables 18 figures
Appendix : Section S8	current limitations	current limitations of our modeling framework that are due to uncertainty and data and model assumptions.	–

### The BEEHAVE model in short

The honey bee model BEEHAVE integrates in‐hive colony dynamics, in‐hive varroa mite population dynamics, mite‐mediated disease transmission, and foraging for nectar and pollen in heterogeneous landscapes. It was designed to explore how various stressors and their interactions affect the structure and dynamics of a single honey bee colony (Becher et al. 2014). BEEHAVE is implemented in the freely available software platform NetLogo (Wilensky 1999). The model, its detailed description following the ODD (Overview, Design concepts, Details) protocol (Grimm et al. 2006, 2020), and a user manual are *available* *online*.^6^ Important model assumptions and equations are listed in Appendix S1: Tables S1, S2.

BEEHAVE's colony module is based on age cohorts and describes, on a daily basis, in‐hive colony structure and dynamics driven by the queen's egg‐laying rate, weather, and forage input. The bees' developmental stage and disease status, available nursing bees, and the colony's honey and pollen stores determine the mortality rates of brood and adult bees. BEEHAVE's foraging module is individual‐based and represents the bees' foraging behavior; it is executed once per day and operates on a time scale of minutes. Weather conditions affect the daily time allocated for nectar and pollen collection. Landscape features, including changes in spatiotemporal availability of nectar and pollen can be updated every day.

In‐hive dynamics and foraging are linked via energy and protein budgets: foragers, in‐hive bees, and brood require certain amounts of energy and protein provided by nectar and pollen, respectively. These requirements are satisfied with incoming forage and are linked to the production and consumption of nectar and pollen stores. A work force that is too small to care for brood can lead to reduced brood production. The distance of flowering patches to a colony and their nutritional reward determine the energetic efficiency of foraging, which is communicated within the colony via a representation of the waggle dance (Appendix S1: Table S2).

### Initial settings

Following BEEHAVE's default settings, all simulations started on 1 January with an initial colony size of 10,000 worker bees and 25 kg honey, which is the amount of honey needed to let a colony of this size survive until spring. As this study focuses on impacts of farmland structure and crop composition, we considered neither virus‐transmitting varroa mite infestation nor its management. Previous simulations found that, with an untreated varroa infestation, 50% of model colonies died after 4 yr even under beneficial foraging conditions (continuous forage supply and 500 m flight distance). Increasing flight distances to the forage patch accelerated colony failure, but the increasing prevalence of the virus in the colony over time in declining bee population had a stronger effect (Becher et al. 2014). Effects of pesticide exposure have been studied elsewhere (Rumkee et al. 2015, Thorbek et al. 2017, Schmolke et al. 2019), and the effects of combined stressors are demonstrated by Henry et al. (2017). Weather conditions define the maximal daily foraging period. We chose the annual Rothamsted (2011) weather option already provided in the BEEHAVE model (based on a data set from Rothamsted, UK, from 2011) for all scenarios and for each of the simulated years, as it offers favorable foraging conditions from early spring till late autumn without prolonged periods of bad weather to avoid interference with forage gaps (Appendix S1: Fig. S1).

As phenological data on species‐specific nectar and pollen availability are scarce, we assumed crop‐species‐specific nectar and pollen contents per flower and sugar concentration to be constant over the corresponding flowering period. To represent early spring foraging on days when weather conditions are suitable for foraging flights, we placed a forage patch providing low daily amounts of nectar (1 L) and pollen (0.5 kg) from January until end‐March at 1,000 m from the hive, which allows the colony to survive the early spring period (Horn et al. 2016). This forage patch represents early flowering plants such as snowdrop (*Galanthus nivalis*), crocus (*Crocus*spp.) and willow trees (*Salix*spp.). The available nectar and pollen amounts at this forage patch can be completely depleted on a given day, so the time a forager needs to collect a full nectar or pollen load (handling time) increases with the degree of forage depletion at this patch.

### Farmland factors for generating cropping system scenarios

**Fig. 1:**
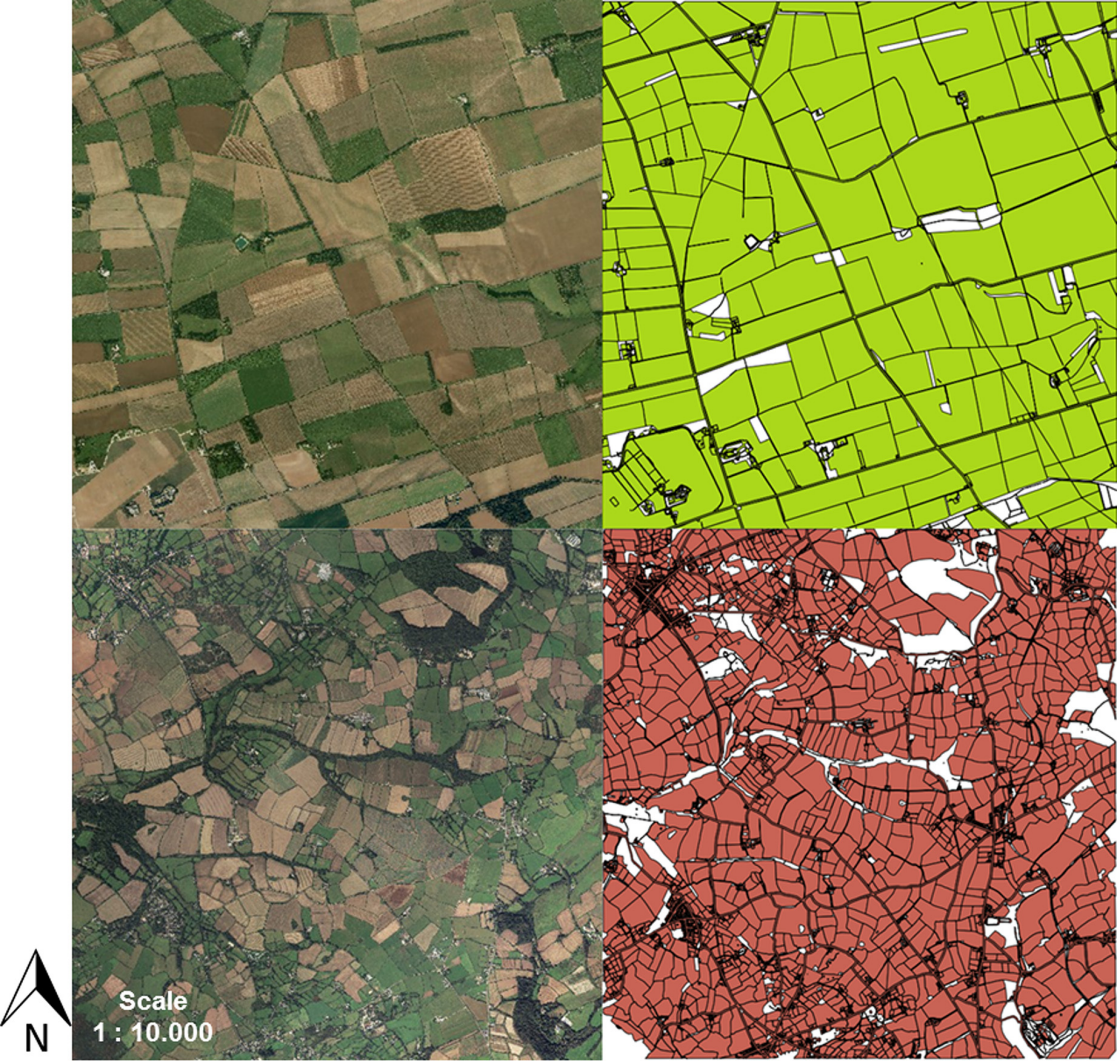
Aerial (left) and raster (right) images of 5 × 5 km maps of the two farmland structures: a simple‐structured farmland located in Lincolnshire, Nocton Heath (above) and a complex‐structured farmland located in Cornwall, Trenwheal (below) (vector data were taken from © OS VectorMap Local 2014). These farmland structures are used as templates to generate various scenarios of cropping systems differing in their crop composition and landscape configuration. White and black areas represent buildings, roads, small forested areas, and hedgerows and were not considered.

**Fig. 2:**
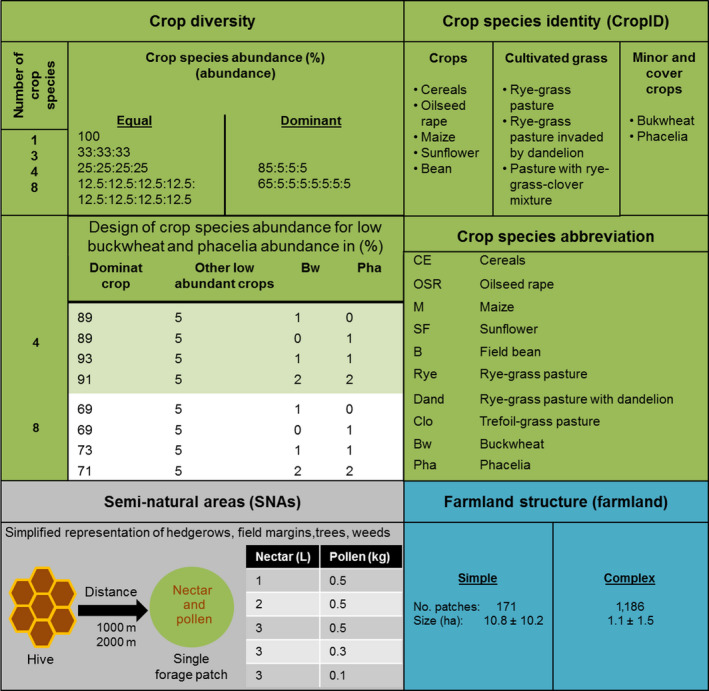
Design of cropping system scenarios. Our design consists of the following variables: number of crop species (#Crops), crop species abundance (in %), crop species identity (cropID), and farmland structure (farmland; simple vs. complex), and representation of forage‐providing semi‐natural areas (SNAs). Representations of SNAs are simplified in terms of one single patch of 1 ha size providing nectar amount from 1 up to 3 L and pollen from 100 to 500 g on a daily basis. This single seminatural habitat patch providing continuous forage over the whole year was located 1,000 or 2,000 m away from the hive. Abbreviations of crop species used in the text and the following figures and tables are given.

We investigated various aspects of cropping systems in terms of farmland structure, crop species identity, and crop diversity represented by number and relative abundance of different crop species (Figs. 1, 2). We made simplified assumptions about crop rotation and the distribution of seminatural habitats in agricultural systems as data about floral phenology, pollen and nectar quantities and qualities of plant species of seminatural habitats are scarce (Baude et al. 2016).

#### Farmland structure

We used 5 × 5 km maps of two farmlands differing in their spatial structure (hereafter called “farmland structure”) as templates for our semi‐realistic landscape settings (Fig. 1). As one landscape, we chose an agricultural region with many small agricultural holdings owning small crop fields as common for example in Western Germany and Southwestern England. As second landscape structure, we selected an agricultural region with few agricultural holdings owning large fields as common for example in Eastern England and Eastern Germany. The first landscape was characterized by small field sizes (<1 ha), while the second one by large ones (>4 ha). The maps (© OS VectorMap Local 2014, Ordnance Survey Limited, Southampton, UK) were taken from the Trenwheal area in Cornwall (small fields) and from the Nocton Heath area located in Lincolnshire (large fields, Table 2). Methodological details are given in Appendix S1: Section S2.

**Table 2:** Attributes of both farmland structures.

Structure	Farmland name	OS grid reference	Latitude (°N)	Longitude (°W)	County (in England)	No. patches	Patch size (ha)			Distance to the hive (m)		
							Mean ± SD	Minimum	Maximum	Mean ± SD	Minimum	Maximum
Simple‐structured farmland	Nocton Heath	tf06sw	53.127797	0.50695178	Lincolnshire	171	10.8 ± 10.2	1.1	68.91	2092 ± 768	0.1	3,730.1
Complex‐structured farmland	Trenwheal	sw63sw	50.121444	5.3588701	Cornwall	1,186	1.1 ± 1.5	0.06	13.3	2463 ± 961	35.2	4,613

BEEHAVE does not directly use GIS maps but requires, as input, a list of the size, distance, and availability of forage for all field patches. To identify the size and location of each field patch and its distance to the hive for both GIS farmland maps, we used the software tool BEESCOUT (Becher et al. 2016). For the simple‐structured farmland, 171 field patches were identified with an average size of 10.82 ± 10.21 ha, whereas the complex‐structured one contained 1,186 field patches with an average size of 1.09 ± 1.54 ha (Table 2). We placed the hive of the simulated colony always at the same central location for both maps (Appendix S1: Table S3).

#### Crop species identity and species‐specific nectar and pollen data

In our hypothetical cropping systems, we distinguished 10 crop species (Fig. 2), consisting of common European agricultural crops, forage crops as common in intensive pastures, and important European minor and cover crops (Eurostat 2015, FAO 2015; Appendix S1: Tables S4, S5 and Figs. S2–S13).

As agricultural crop species, we considered cereals (wheat, rye, barley, and oats were pooled together, providing no resources to bees by themselves), oilseed rape (*Brassica napus* L.), maize (*Zea mays* L., providing only low‐nutritional pollen to bees), sunflower (*Helianthuus annuus* L.), and field bean (*Vicia faba* L.). We further considered three different pasture types common in many temperate parts of Europe for grazing, forage harvesting, silage, and hay production (De Vliegher and Carlier 2007). Most pastures are solely sown with rye‐grasses (e.g., *Lolium perenne*), whereas other grasses such as *Dactylis glomerata* are rarely sown (De Vliegher and Carlier 2007, Eurostat 2015). Especially, in permanent pastures, perennial grasses (mainly *Lolium perenne*) combined with white clover (*Trifolium repens*) are preferred (De Vliegher and Carlier 2007). White clover in a trefoil–grass pasture will ensure a high forage quality improving both energy and protein content for livestock (Hall 1993). For the third pasture type, we included long‐lived perennial dandelion (*Taraxacum officinale*) as a valuable forage plant for livestock, as this commonly infests sown rye‐grass pastures from nearby roads and field margins (Gibson 1997).

Further, we implemented important European minor and cover crops. Phacelia (*Phacelia tanacetifolia*) and buckwheat (*Fagopyrum esculentum*) are rapidly growing flowering cover crops in annual cropping systems, grown to improve soil quality and are recommended as bee pasture (Bjorkman and Shail 2010, Decourtye et al. 2010, De Baets et al. 2011, Lee‐Mader et al. 2014). Buckwheat is also an important minor crop grown for grain‐like seeds (Michalova 2001), whereby major producers are France, Ukraine, and Poland (Eurostat 2015, FAO 2015).

To translate the identity of crop species (hereafter referred to as cropID) on a given patch to units of nectar and pollen, we extracted floral data (flowering period, flower density, nectar and pollen volume, sugar concentration) from existing literature. Existing data about the amount of nectar and pollen provided by different crop species are highly variable due to high intraspecific variation, and abiotic and biotic factors affecting productivity of floral rewards (Burkle and Irwin 2009). We thus used average values, which are most likely to be linked with the applied beneficial weather conditions throughout the year (Rothamsted 2011), where flower production is not limited by bad weather conditions. Furthermore, we assumed constant forage provisioning during the flowering period. Overviews of floral data extracted from literature and equations are given in Appendix S1: Tables S6, S7.

**Fig. 3:**
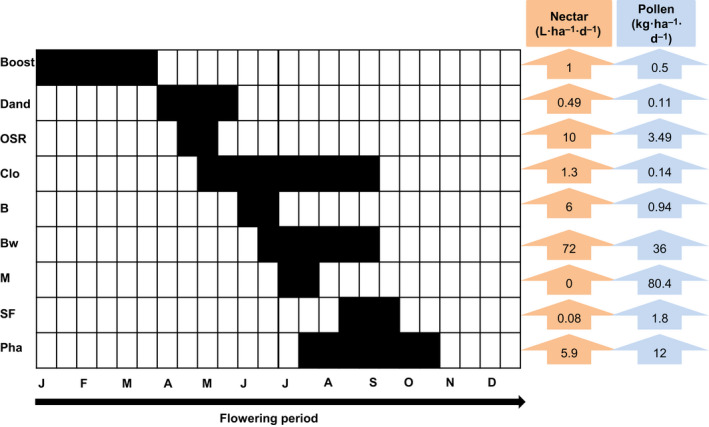
Seasonal flowering phenology of crop species and their average daily nectar and pollen amounts per hectare produced over their specific blooming period. Oilseed rape (OSR) provides on average 10 L nectar and 3.49 kg pollen per day on 1 ha over its blooming period from mid‐April to mid‐May.

We used the flowering period (defined by the start and end days within a year), the amount of nectar and pollen provided per day, and the sugar concentration of each crop species as input data for the honey bee model BEEHAVE. The resulting data of nectar and pollen availability are in the range of nectar productivity for arable land according to Baude et al. (2016; Table 3, Fig. 3). Sensitivity analysis of nectar and pollen quantities and qualities of high‐ and low‐rewarding crop species are available in Appendix S1: Table S14 and Figs. S15–S17.

**Table 3:** Nectar and pollen availability of crop species: flowering period of each crop species is given by its start and end date of blooming within a year.

Crop ID	Flowering period (d)		Nectar (L/m^2^)	Pollen (g/m^2^)	Sugar concentration (mol/L)
	Start	End			
Oilseed rape (*Brassica napus*)	114	144	0.001	0.349	1.7
Maize (*Zea mays*)	197	210	0	8.036	0
Sunflower (*Helianthus annuus*)	237	264	0.000008	0.18	0.7
Field bean (*Vicia faba*)	153	182	0.0006	0.0945	1.46
Cereals	1	365	0	0	0
Dandelion (*Taraxacum officinale*)	92	169	0.000049	0.011	1.19
White clover (*Trifolium repens*)	140	242	0.00013	0.0141	1.08
Rye‐grass (*Lolium perenne*)	1	365	0	0	0
Buckwheat (*Fagopyrum esculentum*)	172	260	0.0072	3.6	1.17
Phacelia (*Phacelia tanacetifolia*)	196 265	255 301	0.00059 0.000443	1.2 0.9	1.05

Note

The amount of nectar and pollen per m^2^ of each crop species is calculated by its mean number of open flower units per m^2^ and its average daily amount of nectar and pollen produced per flower unit (i.e., single floret or flower head). Sugar concentration is given in mol/L. Abbreviations of each crop species is given (these are used in Table 4 and in the Figs. 3, 5). Literature data and equations for calculations are provided in the Appendix S1: Section S3, Tables S6, S7.

Farmlands do, of course, provide certain amounts of nectar and pollen in seminatural habitats. Since very limited data are available on the amount and spatiotemporal distribution of these seminatural resources (but see Baude et al. 2016 and Becher et al. 2018), we ignored them in our overall analyses. However, we estimated how much this simplification affects the results by an in‐depth mechanistic analysis of two scenarios contrasting in crop diversity (a four‐crop and an eight‐crop species combination both resulting in rapid colony extinction). As shown in Fig. 2, we tested different scenarios by introducing a seminatural habitat corresponding to 1 ha in size. We varied the daily nectar amount from 1 up to 3 L and the daily pollen supply from 100 to 500 g in this patch. We applied two flight distances of 1,000 m (intermediate distance as shown for hypothetical landscapes) and 2,000 m (“stressful distance” as shown for artificial landscapes; Appendix S1: Table S15 and Figs. S18, S19; Horn et al. 2016). The assumed nectar amounts for the seminatural habitat are in the range of the estimated yearly nectar productivity per hectare of land‐use classes, e.g., improved grassland or broad‐leafed woodland reported by Baude et al (2016).

#### Crop diversity: number and relative abundance of crop species

We characterized crop diversity by the number of crop species and their relative abundance in the arable land. The number of crop species (hereafter called “#Crops”) considered were: one crop (monocultures), and combinations of three, four, and eight crop species (Fig. 2).

The relative abundance of crop species (hereafter called “abundance”) refers to the proportion of the arable land in the farmland that is occupied by the crop species considered. For four and eight crop combinations, we contrasted *equal* and *dominant* distributions of abundances. For example in the case of four‐crop species, equal meant 25% abundance for each crop species, whereas dominant meant that one crop species occupies most of farmland area, i.e., 85%, and each of the other three 5%. For dominant abundances, each of the crops considered got an equal turn in being the dominant species (Fig. 2).

Additionally, we ran simulations with a low‐abundance treatment of 1% or 2% for buckwheat and/or phacelia in farmland instead of 5%, because these minor and cover crops are more likely to be implemented at very low abundances at the landscape scale in real agricultural systems. For such simulations, the abundance of the dominant crop species, e.g., in a four‐crop combination, was set to 89–93% depending on the low‐abundance treatment (Fig. 2).

### Landscape generator NePoFarm

The input file required by BEEHAVE comprises a list containing the respective data on patch type (i.e., cropID), size, location, detection probability, patch size‐dependent nectar and pollen amounts, and sugar concentrations for each of the farmland patches for each day of the year. To produce input files for different cropping systems in an automated way, we developed a landscape generator, referred to as NePoFarm, implemented in R version 3.1.2 (R Development Core Team 2014), to generate daily nectar and pollen availability lists throughout a year (Appendix S1: Data S1).

### Experimental design and analyses

We ran simulations for 5 yr or until the colony became extinct and used 30 replicates to take into account the stochasticity represented in the model (Becher et al. 2014). Our preliminary sensitivity test indicated that 30 replicates per scenario were sufficient to reduce variation in the outcomes (Appendix S1: Figs. S20, S21).

We explored colony viability for the combinations of farmland structure, crop species identity, and crop diversity listed in Fig. 2. For each of the five simulation years for each cropping system scenario, we used the same input file generated by NePoFarm. Thus, we did not implement crop rotation, for several reasons: crop rotation in a certain farmland area usually only changes the spatial arrangement of certain crops but not their relative proportion and thus not necessarily overall forage availability. For default settings of BEEHAVE, where all fields have a detection probability of one and foragers are thus assumed to cover the entire area modeled, arrangement should play a minor role.

### Indices for quantifying colony viability

#### Colony viability

We quantified honey bee colony viability in terms of the survival probability (hereafter called “SurvProb”; defined as the percentage of replicates in which a colony survived 5 yr) and in terms of the mean time to extinction (hereafter called “TTE”; this metric was used only when the colony survived in none of the 30 replicates). Colonies smaller than 4,000 adult worker bees on 31 December, and colonies where the number of adult bees went down to 0 within the year (e.g., due to depletion of honey stores), were considered extinct or unable to survive without intervention by beekeepers (Becher et al. 2014, Rumkee et al. 2015, Horn et al. 2016).

#### *P*_H_/*P*_P_: indices for comparing forage‐induced stress at the colony level

To compare the effects of forage availability determined by the spatiotemporal nectar and pollen supply of the different scenarios, we calculated new indices: *P*_H_ (honey‐to‐worker‐productivity), and *P*_P_ (pollen‐to‐worker‐productivity). They evaluate how much nectar (L) and pollen (kg) provided by a certain cropping system in a given landscape were converted into the production and maintenance of the colony's work force in terms of worker bees over 5 yr or over the time to extinction. The calculation of these indices includes several parameters about daily pollen and nectar consumption of a single worker bee over its complete larval and adult life span. These are used to calculate the number of worker bees generated as hatched larvae to adults over TTE (*N*_Workers_). To account for different life spans of different bee categories (e.g., short‐living summer bees, long‐living winter bees) we averaged the life span over all these bee categories according to Rortais et al. (2005). In these two indices, we ignored the production of queens and drones and their corresponding food requirements, as they do not directly contribute to the colony's work force. All parameters, equations and cited literature for calculation of these indices are given in Appendix S1: Table S13.

We compared these indices with empirical estimates of an average colony's annual food requirements. According to the literature a viable honey bee colony produces 100,000 up to 200,000 workers per year and thus requires an annual supply of 48–80 kg honey and 20–55 kg pollen (Seeley 1985, 1995, Keller et al. 2005). We applied the lower bounds as the minimum threshold for colony viability according to Seeley (1995) for comparing *P*_H_ and *P*_P_ derived from our simulations with the empirical estimates of viable colonies. Accordingly, *P*_H_ and *P*_P_ values from our simulations have to exceed 48 kg honey and 20 kg pollen, respectively, per year, summed up to a minimum conversion of 240 kg honey and 100 kg pollen over the 5 yr simulation time, to achieve long‐term colony viability.

To qualitatively rank farmland factors by their importance for colony viability in terms of TTE, *N*_Workers_, *P*_P_, and *P*_H_, we applied a procedure mimicking a local sensitivity analysis by varying only one factor at a time while the others were fixed at their nominal values (Saltelli et al. 2000). Details about this procedure are given in Appendix S1: Section S6.

### Effects of forage supply of different cropping systems on colony dynamics

Scenarios composed of four and eight crop species, which showed contrasting impacts on colony viability and *P*_H_ and *P*_P_, were selected to explore in more detail the effects of spatiotemporal forage supply on colony dynamics and resilience. We selected scenarios where (1) the colonies died quickly within the first year (hereafter referred to as “bad forage supply”) and (2) the colonies survived over the simulation time of 5 yr (hereafter referred to as “continuous forage supply”). To understand how these scenarios affected colony dynamics, we analyzed the frequency, timing and duration of forage gaps, i.e., periods in which honey bees cannot find any nectar and pollen in the landscape. Additionally, we wanted to understand whether in these selected scenarios the honey bee colony is able to satisfy its forage demand to maintain, after a disturbance (in this case foraging gaps), the basic functionality of the colony in terms of colony's structure and dynamics, i.e., the life stages and tasks. We focused, following Horn et al. (2016), on mortality effects caused by a lack of pollen (quantified by the proportion of larvae that died in a model step, a day, because of lack of protein from pollen, referred to as “% larval losses: pollen lack”), reduction in the queen's egg‐laying rate (daily number of eggs by which the potential egg‐laying rate was reduced, referred to as “# eggs not laid”), the number of larvae and worker bees, which determines the brood to nurse bee ratio (daily number of all larval stages and all adult workers, referred to as “# larvae” and “# workers”), flight trips (daily number of flight trips to search for forage resources and to forage for nectar and pollen), and stores (daily amount of honey stored in kg, referred to as “honey [kg]”).

## Results

### Indices for quantifying colony performance

#### Colony viability: TTE and SurvProb

**Fig. 4:**
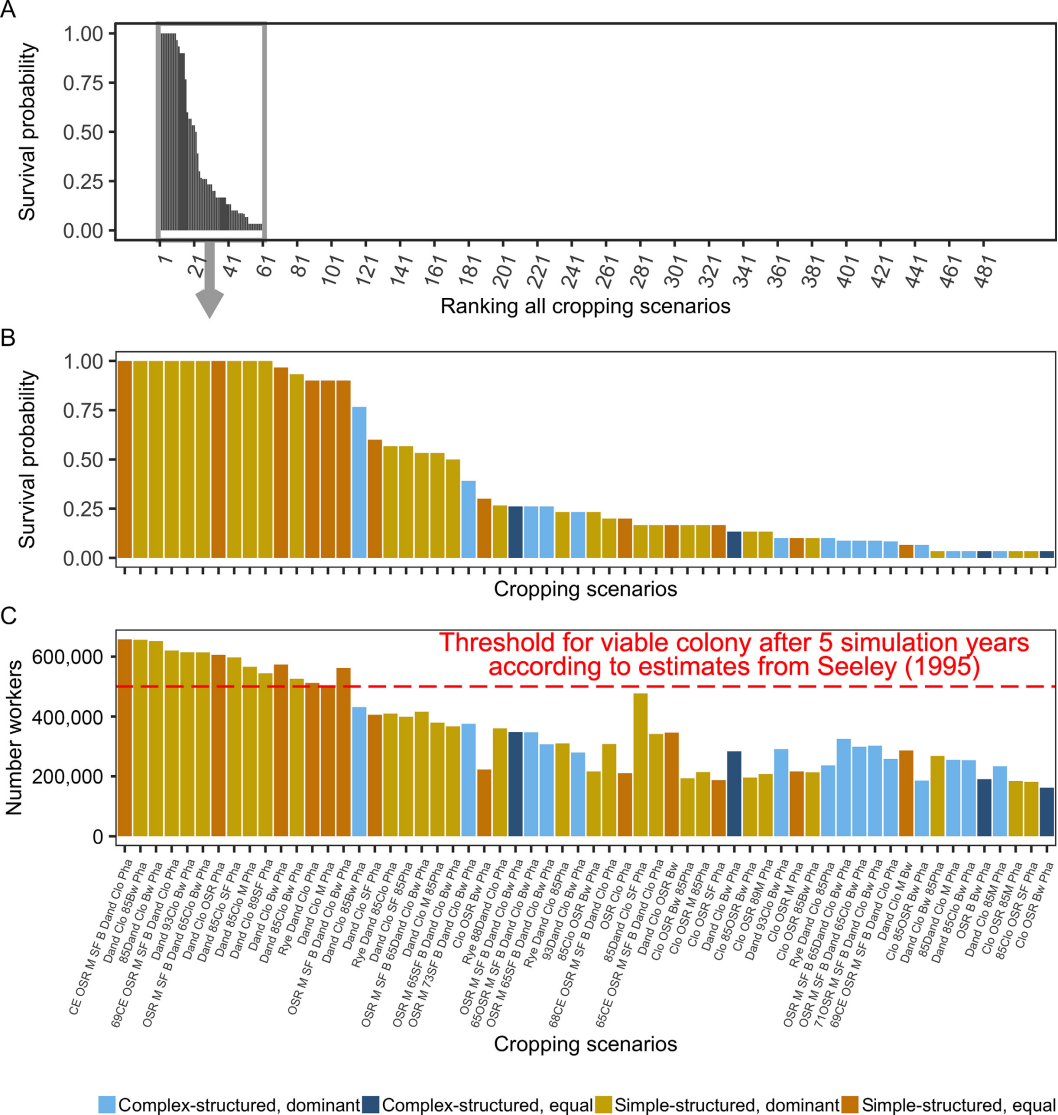
Ranking cropping system scenarios: (A) SurvProb (survival probability) of all screened scenarios over the five simulation years. (B) SurvProb of 60 out of 498 scenarios. The *x*‐axis indicates the crop composition of the scenarios with colony in at least one, out of 30 replicates, surviving for 5 yr. (C) *N*_Workers_ (number of workers produced until the colony collapse or the end simulation time) for the scenarios with at least one surviving colony over the simulation time of 5 yr. The threshold of produced workers for a viable colony after 5 yr of simulation is marked by the red dotted line. Abbreviations of the crop species are given in Fig. 2.

In total, we screened 500 cropping system scenarios resulting in 60 with SurvProb > 0, i.e., at least one colony out of the 30 replicates was still alive after 5 simulation years (Fig. 4). In total, we ran 15,000 simulations, in which colonies survived in 10.3% of all the scenarios with eight crop species and in 4.3% in the scenarios with four‐crop species. For all monocultures, the colony went extinct within the first year. These, and further results are summarized in Table 4.

**Table 4:** *N*_Workers_, *P*_P_, and *P*_H_ over 5 simulation years or until the colony became extinct, and time to extinction (TTE) for selected cropping system scenarios.

Cropping system scenario	Spatial structure	*N*_Workers_	*P*_P_ (kg)	*P*_H_ (kg)	TTE (yr)
Monocultures					
OSR	Simple	33,074 ± 1839	6.6 ± 0.4	16.4 ± 0.9	0.7 ± 0
Ce	Simple	25,736 ± 1038	5.1 ± 0.2	12.7 ± 0.5	0.5 ± 0
Pha	Simple	25,736 ± 1038	5.1 ± 0.2	12.7 ± 0.5	0.5 ± 0
Cropping systems of four‐crop species					
Ce_OSR_M_SF_equal	Simple	41,200 ± 1689	8.2 ± 0.3	20.4 ± 0.8	1 ± 0
Ce_85OSR_M_SF	Simple	40,617 ± 2039	8.1 ± 0.4	20.1 ± 1	1 ± 0
85Ce_OSR_M_SF	Simple	40,255 ± 2347	8 ± 0.5	19.9 ± 1.2	1 ± 0
Ce_85OSR_M_SF	Complex	40,047 ± 2024	7.9 ± 0.4	19.8 ± 1	1 ± 0
85Ce_OSR_M_SF	Complex	40,057 ± 1744	7.9 ± 0.4	19.8 ± 0.9	1 ± 0
Ce_OSR_M_SF_equal	Complex	39,891 ± 1847	7.9 ± 0.4	19.7 ± 0.9	1 ± 0
Dand_Clo_89SF_Pha	Simple	544,372 ± 64,059	108 ± 12.7	269.5 ± 31.7	>5
Dand_Clo_SF_Pha_equal	Simple	406,340 ± 66,026	80.6 ± 13.1	201.1 ± 32.7	4.8 ± 0.5
Dand_Clo_SF_Pha_equal	Complex	212,997 ± 43,475	42.3 ± 8.6	105.4 ± 21.5	3.1 ± 0.7
89Dand_Clo_SF_Pha	Complex	207,940 ± 38,926	41.2 ± 7.7	102.9 ± 19.3	2.9 ± 0.6
Dand_Clo_89SF_Pha	Complex	178,148 ± 28,451	35.3 ± 5.6	88.2 ± 14.1	2.5 ± 0.5
89Dand_Clo_SF_Pha	Simple	113,615 ± 8636	22.6 ± 1.7	56.2 ± 4.3	1.0 ± 0
Cropping system of eight crop species					
Ce_65OSR_M_SF_B_Rye_Dand_Clo	Simple	197,654 ± 31,824	39.2 ± 6.3	97.8 ± 15.8	2.2 ± 0.4
Ce_OSR_M_SF_B_Rye_65Dand_Clo	Simple	138,203 ± 16,078	27.4 ± 3.2	68.4 ± 8	1.4 ± 0.3
Ce_OSR_65M_SF_B_Rye_Dand_Clo	Simple	126,210 ± 11,910	25 ± 2.3	62.5 ± 5.9	1.2 ± 0.3
Ce_OSR_M_SF_B_Rye_Dand_Clo_equal	Simple	131,381 ± 8914	26 ± 1.8	65.0 ± 4.4	1.1 ± 0
Ce_OSR_M_SF_B_Rye_Dand_Clo_equal	Complex	107,585 ± 7037	21.3 ± 1.4	53.3 ± 3.5	1 ± 0
Ce_OSR_65M_SF_B_Rye_Dand_Clo	Complex	106,247 ± 8447	21.1 ± 1.7	52.6 ± 4.2	1 ± 0
Ce_65OSR_M_SF_B_Rye_Dand_Clo	Complex	103,526 ± 8136	20.5 ± 1.6	51.2 ± 4	1 ± 0
Ce_OSR_M_SF_B_Rye_65Dand_Clo	Complex	91,896 ± 5215	18.2 ± 1.0	45.5 ± 2.6	1 ± 0
Ce_OSR_M_SF_B_Dand_Clo_Pha	Simple	657,797 ± 33,587	130.5 ± 6.6	325.6 ± 16.6	>5
69Ce_OSR_M_SF_B_Dand_Clo_Pha	Simple	620,675 ± 42,680	123.1 ± 8.5	307.2 ± 21.1	>5
69Ce_OSR_M_SF_B_Dand_Clo_Pha	Complex	258,315 ± 60,594	51.3 ± 12.0	127.9 ± 30	3.6 ± 0.8
Ce_OSR_M_SF_B_Dand_Clo_Pha	Complex	217,462 ± 37,865	43.1 ± 7.5	107.6 ± 18.7	3.1 ± 0.6
Ce_OSR_M_SF_B_65Dand_Clo_Pha	Complex	202,100 ± 38,442	40.1 ± 7.6	100.0 ± 19	2.8 ± 0.6
Ce_OSR_M_SF_B_65Dand_Clo_Pha	Simple	193,275 ± 27,625	38.3 ± 5.4	95.7 ± 13.7	2.1 ± 0.4

Note:

Abundances are assigned as follows: equal (all crops have the same abundance on total agricultural area [TAA]), 85 or 89 (in a four‐crop composition the dominant crop occupies 85% or 89% of TAA), 65 or 69 (in an eight crop composition the dominant crop occupies 65% or 69% of TAA).

#### *P*_H_/*P*_P_ indices

**Fig. 5:**
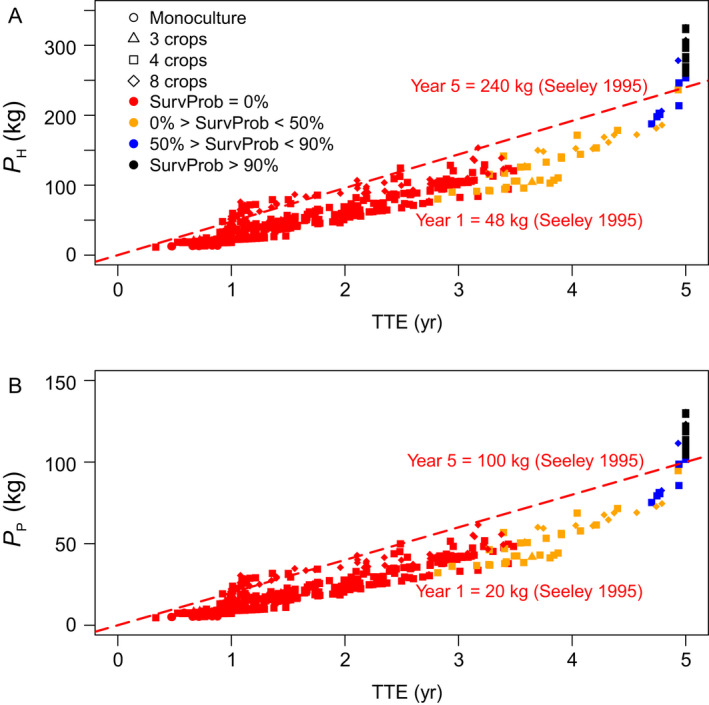
(A) *P*_H_ and (B) *P*_P_, the amount of honey and pollen that the colony was able to convert into production and maintenance of an adult worker bee population in a given agricultural landscape, over simulation time of 5 yr or until the time to extinction (TTE) for all scenarios are plotted. Red dashed lines illustrate empirical estimates of the minimum colony requirements of honey and pollen per year to produce a viable worker population according to Seeley (1985) (at least 48 kg honey and 20 kg pollen per year to produce a sufficient force of 100,000 up to 200,000 worker bees per year). To survive over 5 yr simulation time, a viable colony needs to convert at least 240 kg honey and 100 kg pollen. #Crops is illustrated by symbols and SurvProb by color palette.

Only for scenarios with SurvProb > 0.9 did *N*_Workers_ exceed the threshold of 500,000 adult workers bees (100,000 bees per year summed up over 5 yr) over the entire simulation time, reflecting a viable colony according to empirical estimates by Seeley (1995) as shown in Fig. 4. Fig. 5 shows the achieved *P*_H_ and *P*_P_ values for all scenarios and compares them with minimum thresholds of a viable honey bee colony derived from empirical estimates. For all monocultures, the colony converted up to 16.4 ± 1.2 kg honey and up to 6.6 ± 0.4 kg pollen (mean ± SD; *n* = 30 replicates), which are below the minimum threshold and the colony became extinct within the first year (Table 4). For some scenarios containing four or eight crop species *P*_H_ and *P*_P_ values slightly exceeded the minimum thresholds in the first, up to the third, year (Fig. 5A, B), but colonies were then not able to convert sufficient nectar and pollen into a viable adult worker population thereafter and became extinct within spring or summer of the second or third year (SurvProb = 0).

**Fig. 6:**
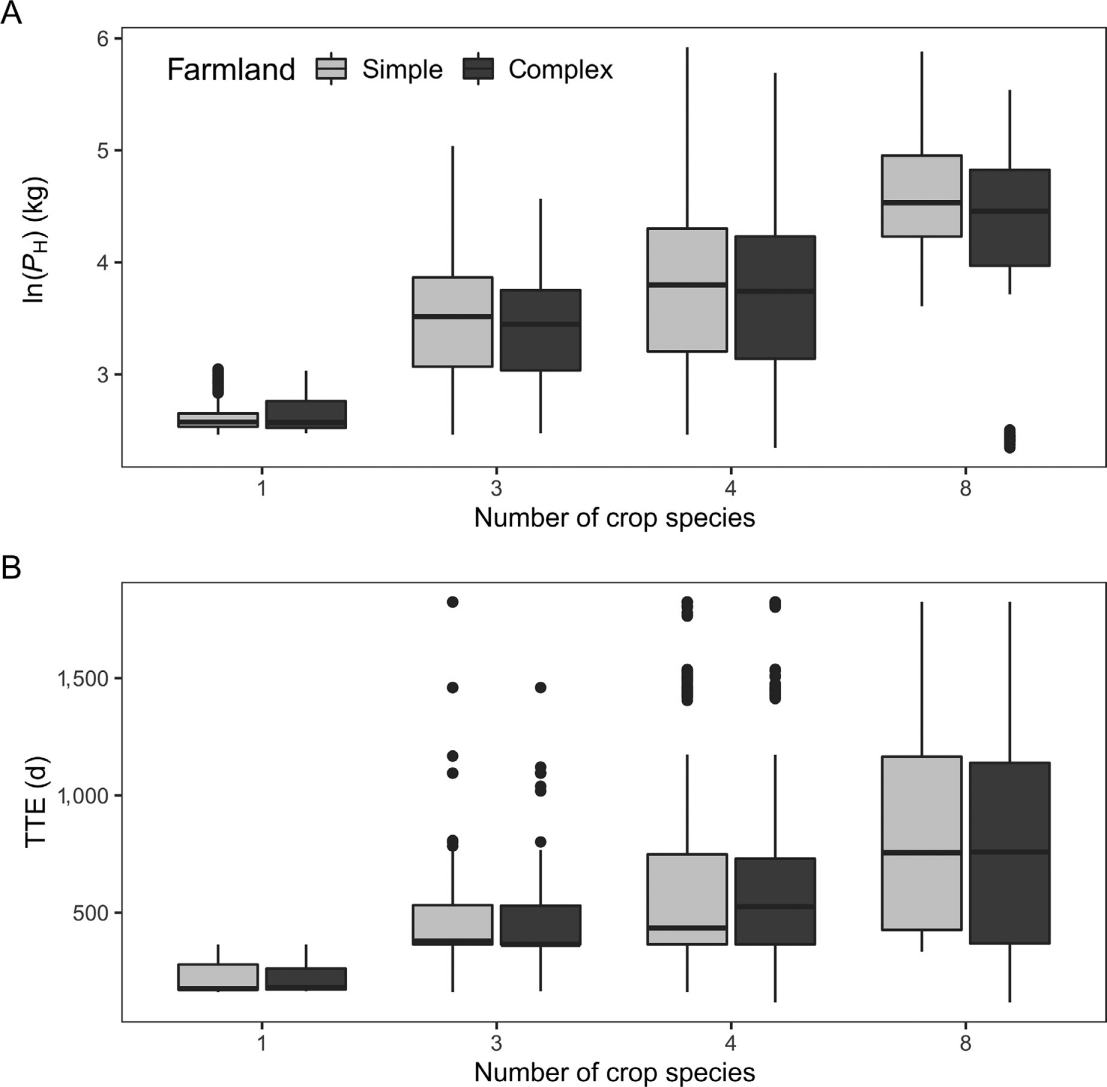
Relation between increasing #Crops and (A) TTE (d) and (B) P_H_ (log‐transformed, kg) for both farmland structures (simple‐structured with few large fields and complex‐structured landscape with many small‐sized fields).

Considering all simulations, increasing *#*Crops led to an increase in TTE and *P*_H_, while differences in TTE between both farmland structures were small (Fig. 6). Our sensitivity analysis indicates that #Crops explains most of the variation in TTE and *P*_H_ ( $R_{\text{adjusted}}^{2}$: 0.119 and 0.3379, respectively), followed by cropID ( $R_{\text{adjusted}}^{2}$: 0.1076 and 0.1363), abundance ( $R_{\text{adjusted}}^{2}$: 0.02078 and 0.02349), and farmland structure ( $R_{\text{adjusted}}^{2}$: 0.003716 and 0.009257). Successful cropping system scenarios frequently included trefoil–grass pastures, phacelia, rye‐grass pastures infested by dandelion, oilseed rape, and buckwheat as crops (Table 4). Moreover, 68.3% of scenarios implemented in simple‐structured landscapes with few, big patches achieved SurvProb > 0, whereas only 31.7% of scenarios implemented in landscapes with many small patches resulted in colony survival (Table 4).

### Effects of forage supply on colony dynamics

#### Cropping system scenarios composed of four‐crop species

##### 1 Four‐crop species: discontinuous, bad forage supply (Figs. 7, 8)

**Fig. 7:**
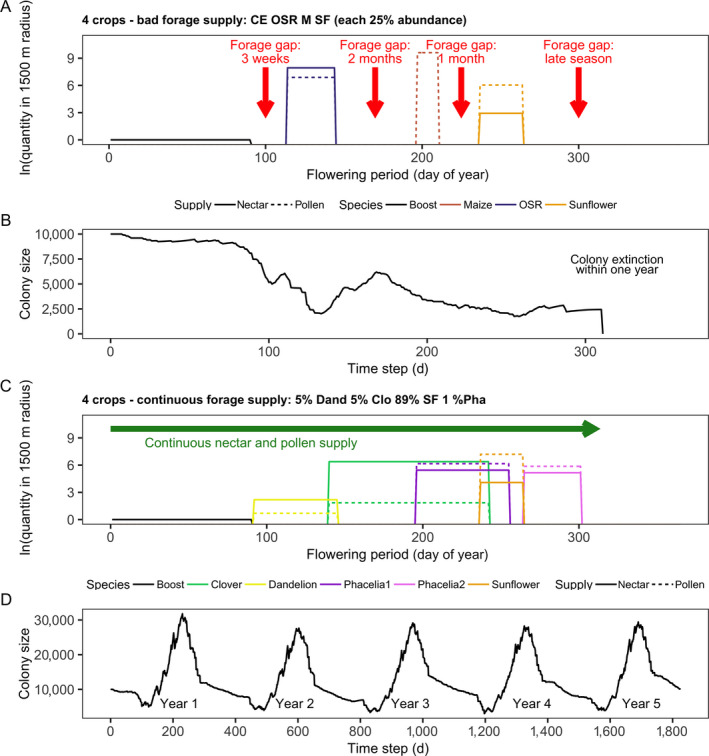
For cropping systems of four crop species: (A, C) seasonal nectar and pollen availability within 1,500 m foraging distance (both log‐transformed; nectar is given in L (black dotted lines; pollen is given in kg [gray dotted line]) and (B, D) honey bee colony size (number of workers) development over time on a daily basis. Frequency, timing, and duration of temporal gaps in nectar and pollen supply (forage gaps) are indicated by red arrows, whereas green arrows indicate continuous nectar and pollen supply over time without forage gaps. Panels A and B demonstrate the results for a bad forage supply and C and D for continuous forage supply.

For scenarios involving the four‐crop species cereals, oilseed rape, maize, and sunflower, for all combinations of abundances and farmland structures, the colony became extinct within the first year (Table 4). As illustrated in Fig. 7A, after the early spring flowering, there was a gap in nectar and pollen supply (hereafter referred to as “forage gap”) of three weeks from the end of March onward before oilseed rape began to bloom in late April. Between mass flowering of oilseed rape until 24 May and the start of maize flowering from mid‐July to the end of July, there is a second and very long forage gap of two months. Then, after the two weeks of pollen provision by maize, from the end of July onward, no nectar and pollen was available for one month before sunflower began blooming in late August (third forage gap). From 22 September onward, nectar and pollen is no longer provided as sunflower ceased blooming (fourth late‐seasonal forage gap).

**Fig. 8:**
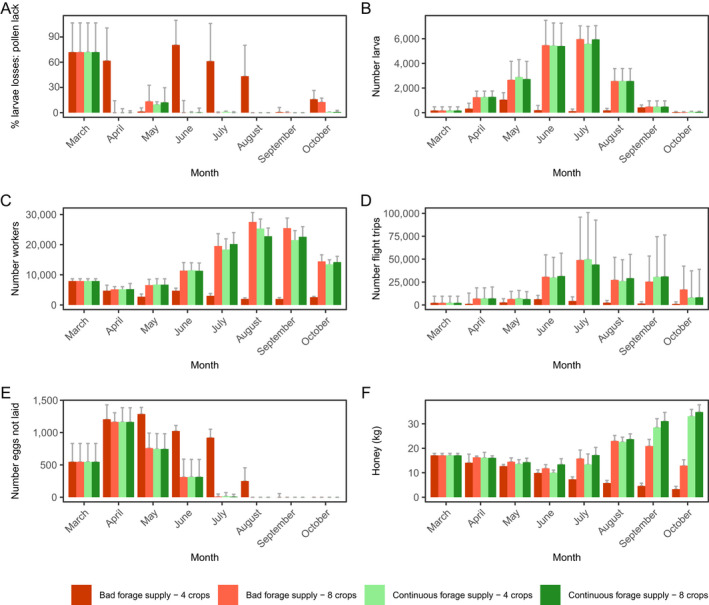
Selected cropping system scenarios composed of four and eight crop species (simple‐structured)—colored bars match scenarios illustrated in Figs. 7, 10: Effects of frequency, timing and duration of temporary gaps in nectar and pollen supply (“forage gaps”) on colony's underlying mechanisms are shown: monthly averages of (A) mortalities of larvae caused by a lack of pollen (proportion of larvae that died in a day due to lack of protein from pollen; “% larval losses: pollen lack”), (B) the total number of larvae and (C) worker bees (daily number of larval stages and adult workers; “# larvae” and “# workers”), (D) flight trips (daily number of searching and foraging trips), (E) reduction in the queen's egg‐laying rate (daily number of eggs by which the potential egg‐laying rate was reduced; “# eggs not laid”), and (F) colony's honey stores (daily amount of honey stored in kg; “Honey [kg]”). Means and standard errors are shown for 30 replicates per scenario.

During the first forage gap, the colony faced high daily larval losses in April due to lack of protein, which led to reduced daily production of later larval stages, and workers in terms of in‐hive bees, and foragers in May (Fig. 8A–C). During the second forage gap, after 6 d without pollen income, the pollen stores were depleted on 1 June. Although no nectar and pollen was available in the landscape, the colony performed more than 4,000 flight trips per day in June and July (Fig. 8D), leading to increased forager mortality. Simultaneously, average daily larval losses and the reduction in the egg‐laying rate of the queen were high in June and July, and few larvae survived (Fig. 8A, B, E). After the third forage gap, sunflower fields provided considerable amounts of nectar and pollen, but the reduced worker force was unable to perform sufficient foraging trips, and honey stores were almost depleted (Fig. 8C, D, F). Consequently, the colony size and its honey stores kept decreasing (Figs. 7B,8C, F). Taken together, colony size rapidly decreased during the year until the colony became extinct at the end of the first year (Fig. 7B).

**Fig. 9:**
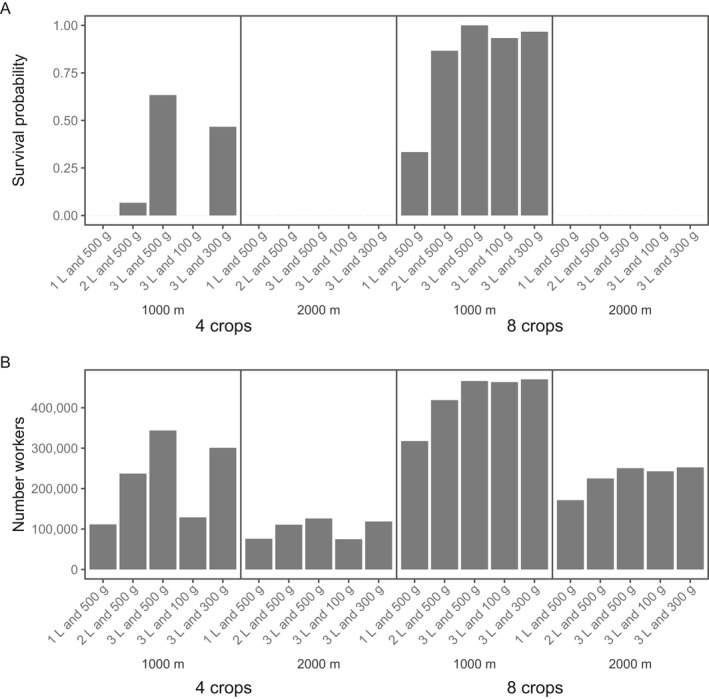
Effects of semi‐natural habitats for two exemplary cropping systems of low diversity (four crops—CE OSR M SF) and high diversity (eight crops—CE OSR M SF B Rye Dand Clo) on (A) survival probability after five simulation years, and (B) colony productivity measured as the number of worker bees produced over five years or until the colony became extinct (*N*_Workers_). Semi‐natural habitats are represented as single forage patches of 1 ha size located 1,000 or 2,000 m away from the honey bee hive and providing constant nectar and pollen rewards over the whole year. The colonies go extinct within one (low diversity example) or two years (high diversity example).

For the scenarios in which we represented floral resources in seminatural areas, colony survival strongly depended on the distance of that seminatural habitat to the hive, which represents the energetic efficiency of exploiting it. If such floral resources provided at least 3 L nectar and 300 g pollen per day throughout the year and were located 1,000 m away from the hive, up to 62% of the colonies were able to survive for 5 yr, but *N*_Workers_ and *P*_H_ and *P*_P_ were below the viability thresholds. Increasing the distance of the additional seminatural floral resources to 2,000 m led to colony extinction in the second year at the latest (Fig. 9).

##### 2 Four‐crop species: continuous forage supply (marked by green bars in Figs. 7, 8)

Scenarios composed of the four crop species: sunflower, rye‐grass pasture infested by dandelion, trefoil–grass pasture, and phacelia provided continuous nectar and pollen supply until the end of October (Fig. 7C). For abundances of 89% sunflower, 5% of each rye–grass pasture infested by dandelion and trefoil–grass pasture, and 1% phacelia, the highest values for *P*_H_ and *P*_P_ were achieved (Figs. 4, 5, Table 4). Here, the abundance of phacelia on the total agricultural area is small, but the patch providing late‐seasonal nectar and pollen supply was placed, by chance, directly adjacent to the hive (0.1 m distance). In scenarios where rye‐grass dandelion or trefoil–grass pastures dominated this cropping system (89%), the few phacelia patches were, by chance, much further away from the hive (>1,806 m), resulting in much lower values for *P*_H_, *P*_P_, and *N*_Workers_ (Table 4). Continuous forage supply, and especially high abundance of nectar and pollen from end‐August until late October close to the hive (89% sunflower, phacelia: adjacent to the hive) as shown in Fig. 7C led to low larval losses from April to August, low reduction in the queen's egg‐laying rate, and a high storage of honey in October (Fig. 8A–F).

#### Cropping system scenarios composed of eight crop species

##### 1 Eight crop species: bad forage supply (marked by orange bars in Figs. 4, 8)

**Fig. 10:**
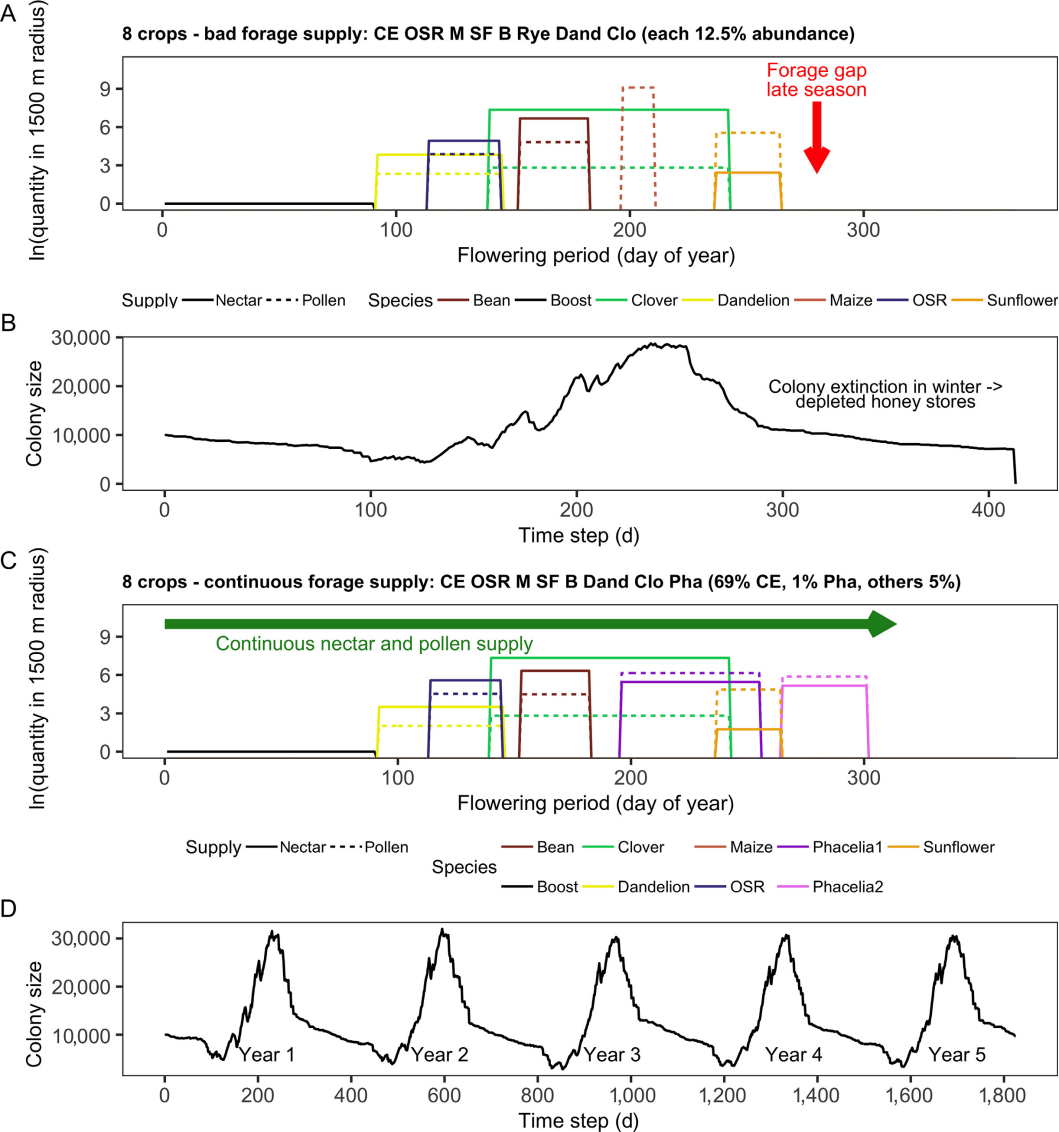
For cropping systems of eight crop species shown are: (A, C) Seasonal nectar and pollen availability within 1,500 m foraging distance (both log‐transformed; nectar is given in l (black dotted lines; pollen is given in kg [grey dotted line]) and (B, D) honey bee colony size (number of workers) development over time on a daily basis. Frequency, timing and duration of temporal gaps in nectar and pollen supply (forage gaps) are indicated by red arrows, whereas green arrows indicate continuous nectar and pollen supply over time without forage gaps. (A) and (B) demonstrate the results for a bad forage supply and (C) and (D) for continuous forage supply.

Cropping systems composed of the eight crop species cereals, oilseed rape, maize, sunflower, bean, rye‐grass pasture, rye–grass pasture infested by dandelion, and trefoil–grass pasture (pasture sown with mixture of rye‐grass and white clover) led to colony extinction within the second year (Table 4). Independent of abundance, dominant crop species and farmland structure in all those scenarios, the cropping system was not able to continuously provide sufficient nectar and pollen to ensure colony viability over the annual cycle (Fig. 4A, B). The cropping system provided continuous forage supply from early spring to mid‐September, but after sunflower ceased blooming, there was a late‐seasonal forage gap from mid‐September onward (Fig. 10). Thus, from mid‐September onward, there were many searching trips in late September and October, but due to the gap in nectar supply, the honey stores decreased in October (Fig. 8D, F).

Again, we ran simulations with additional floral resources of seminatural habitats. If this additional food source provides at least 2 L nectar and 300 g pollen per day, and is located at either 1,000 and 2,000 m distance, it is sufficient to compensate for the late‐seasonal gap and to ensure viability of the modeled honey bee colony. However, colony size and colony honey stores on 31 December were low (1,000 m: 10,841 workers and 11.9 kg honey at the end of the first year; 5,816 workers and 10.3 kg honey after 5 yr), indicating these colonies would not cope well with further stressors such as diseases, pesticide effects, or bad weather.

##### 2 Eight crop species: continuous forage supply (Figs. 8, 10)

For scenarios including cereals, oilseed rape, maize, sunflower, bean, rye–grass pasture infested by dandelion, trefoil–grass pasture, and phacelia as crop species providing continuous forage supply, 217,462–657,797 worker bees were produced (Table 4), which covers the range from poor to good colony performance and viability. The scenario with 12.5% abundance of all eight crop species did not allow the colony to persist. Moreover, if rye‐grass pasture infested by dandelion dominated (65%) and phacelia abundance was 5%, the colonies became extinct within 3 yr in both simple‐ and complex‐structured landscapes (Table 4). In these cases, late‐season forage‐providing phacelia patches were located between 1,000 and 3,500 m from the hive and did not enable colonies to remain viable. On the other hand, even for scenarios where cereals, which do not provide forage at all, dominated the cropping system (69% abundance) and phacelia occurred at very small abundance (1%) in a simple‐structured landscape, the colony produced more than 600,000 worker bees within 5 yr (Table 4, Fig. 4), because late‐flowering phacelia was located adjacent to the hive and provided plenty of nectar and pollen till late‐October (Fig. 10C).

## Discussion

Forage availability in agricultural landscapes is not just a matter of how much nectar and pollen there is but also of where the forage resources are and how their phenology ensures temporal continuity in nectar and pollen supply. We therefore presented a modeling framework to analyze spatiotemporal nectar and pollen supply maps of different cropping system scenarios. Our results demonstrate that number of crop species and their identity determine the frequency, timing, and duration of temporary gaps in nectar and pollen supply (“forage gaps”) and thereby strongly affect colony performance. Frequent and prolonged forage gaps and in particular late‐seasonal forage gaps lasting several weeks showed detrimental impacts on colony viability via cascading effects on colony's life stages and tasks. In contrast, relative crop abundance and farmland structure were less important under our chosen settings (100% detection probability for all fields in the model landscape).

As a metric for colony performance in a given landscape and cropping system, we used SurvProb and in particular the conversion of honey and pollen into the production and maintenance of a viable adult worker population: *P*_H_, *P*_P_, and *N*_Workers_. We compared these metrics with empirical estimates of colony's honey and pollen requirements as reported by Seeley (1985, 1995; see also Keller et al. 2005), which were used to estimate the minimum number of worker bees that are needed to be produced over the year to ensure colony survival. The minimum values for *P*_H,_ *P*_P_, and *N*_Workers_ for viability obtained in our simulations are in good agreement with the values estimated by Seeley (1995) as shown in Table 4 and Figs. 4, 5. It should be noted that we achieved this agreement without any calibration. We take this as a strong indicator that despite the uncertainty in parameters for BEEHAVE, the modeled honey bee colony dynamics are likely to be structurally realistic enough and offers the opportunity to evaluate cropping regimes and mitigation measurements. Still, validating our quantification of the nectar and pollen provided by a given agricultural landscape requires data and experiments that are not available.

### Crop species number and identity determine temporary gaps in forage supply

Our in‐depth analyses of scenarios with four and eight crop species of low vs. high viability showed that there is no simple and general relationship between crop diversity and honey bee viability. Rather, crop composition and in particular the presence and spatial arrangement of particularly rewarding floral resources such as phacelia can be decisive. The reason for the strong impact of number and identity of crop species is that they determine the temporal continuity of nectar and pollen supply and thus the frequency, timing, and duration of forage gaps during the colony's development cycle.

For monocultures, before and after a short‐term period of massive nectar and pollen supply, long‐lasting forage gaps led to rapid colony extinction within 1 yr (Table 4). Similarly, colonies located in cropping systems with three crops were deemed not viable. For some cropping systems with four crops viability occurred, but was still low. In particular for systems composed of the four‐crop species cereals, oilseed rape, maize, and sunflower; which are typical crops in European agriculture (Appendix S1: Table S4 and Figs. S2–S7), colony survival was not possible (Table 4) because these resulted in four forage gaps in April, June–July, August, and late September (Fig. 7A). These forage gaps have detrimental cascading effects on the colony and lead to its rapid extinction within one year. The forage gaps under this cropping system hit the colony during sensitive phases.

In BEEHAVE, the maximum potential egg‐laying rate of the queen is from mid‐June to mid‐July. Thus, the number of newly emerging brood stages developing into new generations of adult worker bees for nursing and foraging tasks is highest in July and August, when the colony is achieving its peak colony size. However, sufficiently high numbers of worker bees over the full annual cycle are necessary to ensure sufficient pollen stores to feed the larvae with protein‐rich jelly. This is required to achieve sufficiently high peak colony sizes and thereby ensure sufficient honey stores to survive over winter (Horn et al. 2016). In BEEHAVE, in accordance with field observations (Blaschon et al. 1999), foragers try to maintain pollen stores that last for about 7 d. After depletion of pollen stores, the larval stages start to die due to decreasing protein content of the jelly. Consequently, flight activity of foragers searching for urgently needed nectar and pollen resources in the surrounding landscape increases.

The first gap of three weeks in April (Fig. 7A) caused high larval mortality due to pollen shortage resulting in lower larval numbers and, consequently, reduced numbers of worker bees in May (Fig. 8). This weakens the colony already in spring. The work force of worker bees for nursing and foraging is thus already reduced before the next prolonged forage gap follows from June to mid‐July (Fig. 7A). Here again, after the pollen stores become depleted, the ratio of foragers to in‐hive bees and flight activity increased. However, as no pollen and nectar is offered within this gap period and as the colony's brood and work force have already been weakened in the April gap, mortality of larvae due to lack of pollen was high and the queen's egg‐laying rate due to lack in available nurse bees was markedly reduced. These losses in this sensitive phase of colony development reduced the number of newly emerging worker bees for foraging and nursing tasks (Fig. 8). The third gap in August (Fig. 7A) worsened the situation. Due to reduced numbers of workers, few foraging trips are undertaken especially in August and September. Thus, honey stores are already very low in August and then the late‐seasonal forage gap (Fig. 7A) deteriorates the situation further (Fig. 8D, F). As a result, honey stores are not sufficient for overwintering and the colony collapses within the first winter.

We conducted additional simulations with representations of additional floral resources that provide continuous but low amounts of nectar and pollen. Whether or not these additional resources are able to buffer the negative effects of forage gaps depends on how energetically efficient it is for the bees to use them. If costs for exploiting these additional resources are low, because seminatural floral resources are nearby, viability could be increased, although not necessarily to the same level as for cropping systems without forage gaps. The effect of seminatural resources is similar to the effect of small but highly rewarding and well‐timed patches of flowering phacelia, which are only efficient if close enough to the hive. These results indicate that seminatural habitats buffer the effects of forage gaps in agricultural systems, but whilst data remains sparse on the quality of resources provided and how efficiently they can be exploited by the bees, it remains unknown as to how well they will buffer the effects of forage gaps in real agricultural landscapes with low crop diversity.

Modeled farmland systems composed of four or eight crop species that covered early‐, mid‐, and late‐seasonal flowering such as oilseed rape or dandelion, and clover or sunflower, and buckwheat or phacelia, even if their overall abundance was low and other common crops such as cereals that do not provide any nectar and pollen dominate the cropping system, were able to offer continuous forage supply (Table 4). These cropping systems therefore ensure forage availability especially during sensitive phases of colony development and ensure long‐term colony viability (Fig. 10).

Continuous nectar and pollen supply without temporary gaps can also be achieved by other flowering crop species than those presented in this study. However, in real landscapes, it might not be sufficient to rely on a limited number of crop species, particularly to ensure pollen nutrition. Pollen varies in terms of availability and concentration of necessary amino acids among plant species, but BEEHAVE neglects such differences in pollen qualities. Dandelion, for example, is important for honey bees in early spring and during times of dearth, but its pollen is lacking in some necessary amino acids resulting in lowered brood rearing success when honey bees rely on dandelion alone (Loper and Cohen 1987). If dandelion (infesting rye‐grass pasture) is the only forage resource in spring, the scope of the model to capture all effects of this reduced pollen quality on brood rearing is limited, which might have led to an overestimation of honey bee colony performance. However, bees tend to collect abundant pollen irrespective of its nutritional value in times when high‐quality pollen is rarely available (Höcherl et al. 2012).

Our interpretation of our results might in principle be limited by having ignored the sampling effect (De Laender et al. 2016): higher diversity scenarios have a higher probability of containing the most nectar‐ and pollen‐rewarding crop species. However, in our case, we are confident we can exclude this effect. The highest rewarding crop species, oilseed rape, buckwheat, and phacelia, occur in 33, 32, and 58 scenarios with SurvProb > 0, respectively, but they were also present in 271 (oilseed rape), 198 (buckwheat), and 254 (phacelia) scenarios without any surviving colony (SurvProb = 0). This, together with similar data for other crop species, makes us believe that our simulations are sufficiently balanced to buffer against sampling effects because the presence of high‐rewarding crops (oilseed rape or buckwheat or phacelia or all three) did not guarantee colony persistence and also for the highest diversity level of eight crop species survival probability is relatively low.

### Effects of spatial arrangement and relative abundance of crops

Overall, both relative crop abundance and farmland structure showed the lowest impact on colony viability. However, details can matter. According to our results, presence of mid‐ and late‐flowering species such as phacelia at low abundance (2% or 1%) can be sufficient to ensure required honey stores for colony survival even in intensively used agricultural systems. For such late‐seasonal floral resources to be effective, they must be located close to the hive. If the distance of these late‐flowering resources increases to >1,500 m from the hive, the foraging efficiency decreases and foragers are not able to gain a sufficient energy surplus if relying on this late‐flowering resource alone.

Thus, in addition to temporal aspects of crop species leading to the presence or absence of forage gaps, spatial aspects also matter: distances of fields with critical crops preventing forage gaps (Table 3) should not exceed honey bees' regular foraging distances as reported by Steffan‐Dewenter and Kuhn (2003) and Couvillon et al. (2014). For example, even if the abundance of phacelia was 5% in cropping system of eight crops, but the fields were too far away from the hive, the colony was not able to satisfy, in the long run, its late‐seasonal demand for nectar to provide honey stores for overwintering (Appendix S1: Tables S15–S23 and Figs. S20–S33 present analyses of stylized landscape where each crop species has just one field, which varies in size and distance to the hive).

One might argue that, in many agricultural landscapes, field sizes larger than our maximum field size, 69 ha, exist. For the single colony represented by BEEHAVE, this does not matter because the forage provided by fields larger than 10 ha cannot be completely consumed by this colony. This figure might change if intra‐ and interspecific competition is considered, but it seems unlikely that changes in the maximum field size considered by us will affect our overall findings.

Crop relative abundance was not important in our simulations, but this might be due to the fact that we did not consider competition with other honey bees and wild pollinators. Mass‐flowering crop fields providing high amounts of nectar and pollen during their blooming period at the landscape scale cannot be depleted by the bees of a single colony as the colony's population of foraging bees is not able to collect all offered nectar and pollen within this short‐term period. Still, even for monocultures of the highest rewarding crop, oilseed rape, maximum patch size (69 ha), and minimum distance (0.1 m) to the hive, colonies collapsed within the first year. This indicates that the long‐lasting gap in forage supply from mid‐May onward after the blooming period of oilseed rape cannot be compensated even by large fields and shortest flight distance of the worker bees to the forage source (additional simulations with simplified, stylized landscapes with three or four‐crop species, are presented in the Appendix S1: Section S7 and provide further mechanistic insight into the consequences of forage gaps.)

Still, even in the case of temporal continuity in the nectar and pollen supply, the spatial arrangement of the rewarding flowers can matter as it determines the energy expenditure by the flying bees. If the energy costs for foraging trips to these flowers exceed the energy gain, the colony in terms of its force of foragers is weakened over time. Longer foraging distances increase forager mortality and reduce production of a new worker generation due to lowered nectar and pollen intake. For example, for a cropping system scenario composed of the eight crops dominated by rye‐grass–dandelion (65% of the agricultural area and all other crops occupy 5%) the colony collapsed in the third year at the latest. Here, none of the five late‐seasonal rewarding phacelia patches is located in a flight distance shorter than 1,000 m.

### Effects of nectar and pollen supply in reality

We demonstrated that we can explain the effects of crop species identity and diversity on colony performance by determining frequency, timing and duration of temporary forage gaps and revealing their impacts on underlying cascading effects on life stages and work force of the modeled colony (Fig. 10). Some of these mechanisms, e.g., reduced number of nursing bees resulting in high larval mortality, are also pointed out by previous modeling studies of honey bee colony dynamics (Rumkee et al. 2015, Torres et al. 2015). Outcomes of this modeling approach reflected some patterns observed in reality, but in general we offer hypotheses regarding colony's response to different cropping systems that can be tested with experiments.

In most temperate zones, seminatural habitats are patchily distributed and sometimes rare in intensively managed agricultural systems. Forage dearth periods occur mainly in summer, but nectar and pollen availability to the bees may vary widely among locations and land‐use types. However, forage supply from weeds and wild plants during gaps heavily depends on their diversity in agricultural landscapes. Our finding that landscape structure is less important than crop diversity and identity is confirmed by field studies that suggest that honey bee foraging is not that much affected by landscape structure as it is by seasonal patterns, whereby especially in summer (June and July) overall forage availability in the landscape is low and is most challenging (Steffan‐Dewenter and Kuhn 2003, Couvillon et al. 2014).

During sensitive phases of colony development, long‐range foraging may weaken colonies because larger foraging distances means higher mortality risk for foraging bees. Therefore, the spatiotemporal dynamics of nectar and pollen in the landscape are important to provide a sufficient energy surplus to the colony (Lonsdorf et al. 2009). Hicks et al. (2016) pointed out that continuous forage availability throughout the year is important for insect pollinator richness and abundances.

Of course frequently occurring forage gaps in agricultural landscapes may lead beekeepers to provide sugar solutions and pollen substitutes, and to move hives to temporary forage‐rich floral resources within the landscape in order to avoid malnutrition and colony collapse. Still, recent studies suggest that decline in honey bee health and hive numbers is due to long non‐foraging periods caused by intensive agriculture at large landscape scales (Brown and Paxton 2009), and insufficient and untimely feeding (Brodschneider et al. 2010).

Our results indicate measures for avoiding forage gaps, especially by implementation of mid‐ and late‐flowering crop species for boosting honey bee colony success in intensive agricultural systems. For instance, reintroduction of clover in legume‐rich grass sward instead of growing “green deserts” or growing phacelia and buckwheat (Decourtye et al. 2010, Woodcock et al. 2015, Sponsler and Johnson 2015), and increasing the abundance and diversity of forage‐rich, seminatural floral resources is needed to overcome temporary gaps in nectar and pollen availability in agricultural landscapes.

### Limitations and next steps

There are a number of limitations in the modeling framework presented here (see also Appendix S1: Section S8). In our simulations, we considered landscapes that are simplified in their crop composition, rotation and abundance of floral resources, detection probability of flowering fields, and in the representation of seminatural habitats. Data about flowering phenology and nectar and pollen rewards for different crop species and plant species of seminatural habitats are scarce and hard to distil from literature and vegetation data bases (Baude et al. 2016). We therefore focused on a selection of the most important crops, pasture types, and minor and cover crops and we assumed constant crop species‐specific nectar and pollen content over the blooming period. Due to the high intraspecific variation in reported data, we used values that are likely to be linked with good weather throughout the year, because the weather data we used (Rothamsted (2011)) represent largely beneficial conditions.

Our results show that even agricultural landscapes of higher diversity may not fulfill the continuous nectar and pollen requirements of the colony over the whole season. Presence of the gaps in forage supply, even if they occur at the end of the foraging season, induce certain levels of stress to the colony but it can take two or more years to accumulate and to completely unfold its consequences to colony's brood stages and work force. This is an important observation from the model to improve bee monitoring for risk assessment.

Furthermore, our simulation study pointed out the important aspect of land‐use patterns, which determines the continuity in forage quantity and quality according to colony's nectar and pollen needs at certain times of development cycle, for risk assessment. In order to include this important driver for bee losses into population models, we need the data for the most important land cover types about floral resources and cover of crops, seminatural habitats, and weeds in high spatial resolution e.g., from remote sensing data on land cover types and plant covers from vegetation data bases or experts. Nectar and pollen data can be derived from quantitative surveys of crops, weed species and plants from meadows, forests and rural habitats. Given that such surveys are labor intensive, pollen and nectar volume can be predicted from statistical models by using flower morphology and flower counts.

Moreover, information about farming practices on the landscape level and regional weather data and forecasts are required in order to predict the floral rewards under several climate conditions. Landscape‐level risk assessment depends on robust information about exposure to pesticides and bee pathogens. Recent modeling studies highlighted that honey bee colonies might be able to tolerate more stress in forage‐rich landscapes and low forage availability makes them more vulnerable to exposure and diseases (Becher et al. 2014, Horn et al. 2016, Thorbek et al. 2017). Parameterization of land‐use pattern‐depended forage intake is therefore an important task to model predictions for risk assessment and management. Agatz et al. (2019) make specific recommendations for collecting data that would allow for further, more detailed validation and, hence, use of BEEHAVE.

BEEHAVE neglects differences in pollen qualities (e.g., amino acids) among different species. In reality, honey bees rely on a high diversity of floral resources to meet their pollen requirements especially in spring. Higher pollen diversity enhances immunity to diseases and tolerance to pesticides (Di Pasquale et al. 2013) and ensures the health and sustainability of the colony. This cannot currently be reflected by the model.

A further important limitation is that we do not consider competition with other honey bee colonies and wild pollinators. Implementing realistic densities and distributions of colonies or apiaries will considerably increase the complexity of an already complex modeling framework, but this challenge needs to be tackled. Regarding competition with wild pollinators, currently available data, except for common bumble bee and solitary bee species, are scarce and need to be reflected via aggregated and conservative assumptions.

In our analysis, we deliberately ignored other stressors such as pathogens, certain beekeeping practices, and pesticide effects. It is therefore important to keep in mind that the predictions of colony performance presented here are relative, not absolute. Certain cropping systems reduce the frequency, timing, and duration of temporary gaps in nectar and pollen supply affecting the colony's life stages and work force and enable higher viability and better performance than others, and this enables the colonies to cope better with additional stress.

## Conclusions

Awareness of the importance of the spatiotemporal continuity of floral resources for many insect pollinators has long been recognized (Vanbergen and the Insect Pollinators Initiative 2013, Sponsler and Johnson 2015, Hicks et al. 2016, Alaux et al. 2017, Dolezal et al. 2019, Guzman et al. 2019), but evidence of landscape context effects on honey bee colony performance and their underlying mechanisms are scant (but see Sponsler and Johnson 2015). Thus, we believe that our modeling framework improves our knowledge of how agricultural systems affect spatiotemporal forage availability and thus the performance of a single honey bee colony. Our most important general outcome is the ability to predict the extent to which forage gaps reveal detrimental cascading effects on colony dynamics and put honey bee colonies at risk, by using the availability of nectar and pollen over time.

Agricultural policies should adopt a way of assessing cropping systems and develop incentives or regulations that minimize forage gaps. These measures should address both cropping systems, and their diversity and composition, and the amount and spatial distribution of seminatural habitats. For representative farmland structures and weather conditions in different eco‐regions (EFSA 2016), the effects of policies and farmland structure and dynamics could be first screened by generating “forage provisioning plots” (Figs. 7A, 10A) and, if in doubt, assessed in more detail by using BEEHAVE.

Our results suggest that increasing crop number increases a colony's viability, but the identity of crop species still determines the frequency, timing and duration of temporary gaps in nectar and pollen supply and thus the strength of cascading effects on the structure and dynamics of a honey bee colony. If the floral diversity of the cropping system does not ensure a continuous supply with nectar and pollen, especially during sensitive phases of colony development, colonies are negatively affected in terms of a reduction in worker bees for nursing and foraging tasks.

Overall, our framework can help to understand, quantify, and rank the effects of different agricultural production systems on honey bee health, to predict colony responses to future anthropogenic land‐use changes, and to test land‐use management strategies and policies on maintenance of honey bees and other pollinators for assurance of pollination services in agricultural systems.

## Supporting information

Appendix S1

Data S1

Metadata S1

## Footnotes

^6^

www.beehave‐model.net
