# Supplementary material for: Honey bee colony performance affected by crop diversity and farmland structure: a modeling framework
Source: Ecol Appl. 2020 Sep 30;31(1):e02216. doi: 10.1002/eap.2216 (PMC11475367; doi:10.1002/eap.2216)
Supplement: Supplementary file 3 — Metadata S1 [file EAP-31-e02216-s001.pdf]

Horn, J., M.A. Becher, K. Johst, P. Kennedy, J.L. Osborne, V. Radchuk, and V. Grimm. 2020. Honey bee colony performance affected by crop diversity and farmland structure: a modeling framework. Ecological Applications.

Data S1

- **R-script implementing the landscape generator "NePoFarm"**
- **Data file (txt format) providing the landscape structure data required by NePoFarm**

Author

Juliane Horn
Helmholtz Centre for Environmental Research-UFZ
Department of Ecological Modelling
Permoserstr. 15
04318 Leipzig
Germany
juliane.horn@uni-goettingen.de

File list (files found within DataS1.zip)

`S2-1-NePoFarm.R`
`S2-2-Input.txt`

Description

`S2-1-NePoFarm.R` – R-script implementing the landscape generator "NePoFarm". A documentation of this script is provided in Appendix S1: Section S4.

`S2-2-Input.txt` – NePoFarm requires as input raster data of all agriculatural fields in two landscapes in England (see Appendix S1: Section S4).
